# Tumor Implantation Site of Syngeneic Oral Cancer Models Differentially Induces Site-Dependent Local and Systemic Immunosuppression

**DOI:** 10.3390/cancers18101607

**Published:** 2026-05-15

**Authors:** Andrea H. Molina, Gemalene M. Sunga, Shawn Nguyen, Neeraja Dharmaraj, Ratna Veeramachaneni, Roberto Rangel, Jeffrey N. Myers, Jeffrey D. Hartgerink, Andrew G. Sikora, Simon Young

**Affiliations:** 1 Katz Department of Oral and Maxillofacial Surgery, School of Dentistry, The University of Texas Health Science Center at Houston, Houston, TX 77054, USA; 2 Department of Head and Neck Surgery, The University of Texas MD Anderson Cancer Center, Houston, TX 77030, USA; 3 Department of Chemistry, Rice University, 6100 Main Street, Houston, TX 77098, USA; 4 Department of Bioengineering, Rice University, 6100 Main Street, Houston, TX 77098, USA

**Keywords:** immune microenvironment, tumor draining lymph node, oral cancer, preclinical models

## Abstract

Background/Objectives: Preclinical studies of head and neck squamous cell carcinoma (HNSCC) commonly use subcutaneous heterotopic (flank) tumor models for simplicity; however, orthotopic models may better reflect the native tumor environment. Direct comparisons of the tumor immune microenvironments (TIME) and tumor-draining lymph nodes (tdLNs) between these models remain limited. Better understanding of site-specific immune differences could improve model selection and interpretation of translational HNSCC studies. Methods: ROC1 tumors were established in murine heterotopic and orthotopic sites, followed by assessment of tumor growth kinetics, survival, and the tumor microenvironment. Immune composition of tumors, blood, tdLNs, and spleen was evaluated at three tumor progression timepoints using multiparameter spectral flow cytometry. Results: Heterotopic and orthotopic tumor models showed similar growth kinetics and survival. Immune profiling revealed increased infiltration of CD3^+^ T-cells, natural killer (NK) cells, and myeloid populations in both models. Heterotopic tumors were enriched in dendritic cells (DCs), plasmacytoid DCs, and monocytic myeloid-derived suppressor cells (M-MDSCs), whereas orthotopic tumors showed increased macrophages, granulocytic MDSCs, and M-MDSCs. Despite temporal variation, both TIMEs were dominated by macrophages, DCs, and CD3^+^ T-cells. Late-stage heterotopic tumors contained more CD4^+^ T-cells. Reduced T-cell cytotoxicity (PD-1, CD107a) and increased immune checkpoint expression across myeloid cells indicated an immunosuppressive TIME. Systemically, effector cells were preserved despite suppressive cell trafficking, and tdLNs in both models exhibited immunosuppressive PD-L1 expression. Conclusions: Heterotopic and orthotopic ROC1 tumors share key immune features, but site-specific differences in the TIME and tdLNs reveal tissue-dependent regulation. These local effects align with systemic changes, supporting global tumor-associated immunosuppression.

## Simple Summary

Preclinical models often use subcutaneous heterotopic (flank) models for the ease of tumor implantation and monitoring. However, orthotopic models are deemed more clinically relevant. Few studies have directly compared the tumor immune microenvironment and associated tumor-draining lymph nodes between orthotopic and heterotopic models. Therefore, we investigated the immune composition of tumors, tumor-draining lymph nodes, blood, and spleen to understand these changes within each tumor model across tumor progression and between models at a late-stage tumor. This study demonstrates that heterotopic and orthotopic ROC1 tumors, despite differing implantation sites, share broadly similar immune compositions while exhibiting distinct, tissue-specific differences in the tumor immune microenvironment and tumor-draining lymph nodes that can influence anti-tumor immune responses. Importantly, it reveals that these local immune differences extend beyond the tumor site to drive systemic tumor-associated immunosuppression.

## 1. Introduction

Most cases of head and neck squamous cell carcinoma (HNSCC) are diagnosed at late stages when treatment is ineffective [1]. HNSCC has high locoregional and distant recurrence rates and can metastasize to the regional lymph nodes in the neck. Tumor excision is commonly performed, with concomitant removal of the tumor-draining lymph nodes when indicated. These surgical procedures, combined with risk-adapted adjuvant chemo- and radiotherapy, are associated with significant morbidity to the patient and may still fail to prevent HNSCC recurrence [2,3,4]. To address these challenges, immunotherapy has emerged as an additional “pillar” of cancer therapy. Immunotherapy is a type of cancer treatment that can program immune cells to target and recognize cancer cells in the primary tumor and the tumor-draining lymph nodes (tdLNs), thereby boosting immunological memory and reducing cancer recurrence. Unfortunately, most patients with HNSCC treated with single-agent immunotherapy do not respond [5], in large part due to the immunosuppressive tumor immune microenvironment (TIME). Therefore, understanding the tumor-specific TIME and immune microenvironment of the tdLNs is crucial to improving the response rates of HNSCC immunotherapies and indicates the need to target cell types that can bridge innate and adaptive immune cell responses. Moreover, current FDA-approved immunotherapies are delivered systemically, so understanding the composition of circulating tumor and immune cells and the potential for long-term immunity by investigating peripheral blood and spleen may further enhance the efficacy of systemic anti-HNSCC response to immunotherapies [6,7].

In cancer research, syngeneic murine models are frequently used to study immunotherapy responses in a functional immune system and interactions in the TIME [8]. Emerging treatments for head and neck cancers are often investigated using subcutaneous heterotopic (flank) models, owing to the simplicity of tumor implantation, ease of tumor monitoring [9] and direct access for treatment such as radiation therapy [10,11]. However, orthotopic models, in which tumors are implanted into the relevant organ-specific environment, are widely considered more translationally relevant, as they more accurately reflect the TIME in humans and predict therapeutic responses [12,13,14,15,16]. Therefore, researchers are developing orthotopic relevant HNSCC models to utilize for treatments [17,18,19,20].

Few studies have directly compared the TIME of tumors in murine models of head and neck squamous cell carcinoma. For instance, preclinical investigations in HPV-positive oral cancer tumors (mEER) established in murine heterotopic (flank) and orthotopic (tongue) models demonstrated site-specific differences in the immune cell composition of the respective tumors. These differences were concluded to impact the resultant protective immunity upon immune checkpoint inhibitor treatment, specifically in the orthotopic model, and, ultimately, the efficacy of single-agent versus combination immunotherapy [21]. Additionally, researchers have compared the TIME and tdLNs in a HPV-negative, immune-infiltrated oral cancer model, mouse oral squamous cell carcinoma cell line 1 (MOC1), to understand the immune cell composition over the course of tumor progression and predict potential immunotherapy responses [22]. mEER and MOC models have been well-characterized, including exploration of tumor site-specific differences [21,22,23]. However, existing models, although frequently utilized, do not fully recapitulate the heterogeneity of head and neck cancer and the human smoking signature. Furthermore, the study of poorly immunogenic models is critical because these are of greatest relevance to the immunotherapy-resistant microenvironment of most head and neck cancers. In this study, we utilized an HPV-negative, poorly immunogenic oral cavity cancer cell line, ROC1. ROC1 is a carcinogen-derived oral cancer cell line that has tobacco mutation signatures relevant to the study of head and neck cancers [24]. The ROC1 line was generated by exposing mice to the carcinogen 4-nitroquinoline-1 oxide (4-NQO), which is reflective of the human smoking signature, and is resistant to traditional immunotherapy [24,25]. To date, differences between orthotopic and heterotopic implantation sites have not been examined in this type of model. We thus hypothesized that there are location-based TIME differences between implantation site and results in a dampened immune response that are extended to the tdLN, peripheral blood and spleen.

This study provides longitudinal, multi-compartment immune profiling of a clinically relevant murine oral cancer model across the tumor microenvironment, tumor-draining lymph nodes, blood, and spleen in both murine heterotopic (flank) and orthotopic (oral cavity) sites of implantation. Understanding the TIME differences between tumors at orthotopic and heterotopic sites will enable researchers to make informed decisions when selecting a tumor implantation site and determine if the head and neck tumor (or tdLN) microenvironment is appropriate to study a specific immunopathological mechanism or immunotherapy.

## 2. Materials and Methods

### 2.1. Murine Tumor Cell Line

The murine oral cancer cell line ROC1 cells were maintained in Dulbecco’s Modified Eagle’s Medium (Thermo Fisher Scientific, Waltham, MA, USA) supplemented with 10% FBS (Cytiva Hyclone Laboratories, Logan, UT, USA), 2 mM L-glutamine (Lonza, Walkersville, MD, USA), 1 mM sodium pyruvate (Cytiva Hyclone Laboratories, Logan, UT, USA), non-essential amino acids (Lonza, Walkersville, MD, USA), 50 U/mL penicillin–streptomycin (Corning, Corning, NY, USA), and an MEM vitamins solution (1X) (Cytiva Hyclone Laboratories, Logan, UT, USA), and incubated at 37 °C in 5% CO_2_. The cells were maintained as described previously [24]. Cells were regularly tested for mycoplasma.

### 2.2. In Vivo Orthotopic and Heterotopic Studies

Six- to eight-week-old wild-type immunocompetent C57BL/6 or Rag1^−/−^ “non-leaky” immune deficient mice, lacking both mature T- and B-cells (Jackson Laboratory, Bar Harbor, ME, USA), were used to establish the tumor models. Tumor size measurements (in diameter) were taken at least 2 times per week to monitor tumor growth using digital calipers (Thermo Fisher Scientific, Waltham, MA, USA) to measure the length and width of the tumor (tumor area). Mice were maintained under standard housing conditions throughout the study.

#### 2.2.1. Establishing the Heterotopic Tumor Model

The ROC1 heterotopic tumor model was established by injecting 100 µL of 500,000 cells in PBS (Cytiva Hyclone Laboratories, Logan, UT, USA) into the subcutaneous space of the murine left flank. The humane endpoint of the heterotopic model was no more than 15 mm in diameter or 225 mm^2^ in tumor area or no more than 20% weight loss.

#### 2.2.2. Establishing the Orthotopic Tumor Model

The ROC1 orthotopic tumor models were established by injecting 30 µL of 500,000 cells in PBS into the maxillary vestibule of the left oral cavity of mice. The humane endpoint of the orthotopic model was no more than 12 mm in diameter or 144 mm^2^ in tumor area or no more than 20% weight loss.

### 2.3. Tissue Processing, Cell Separation, and Data Acquisition

Established ROC1 tumors, tumor-draining lymph nodes, and their respective blood and spleen were collected for flow cytometry when the tumors reached 9 mm^2^ (timepoint 1), 25 mm^2^ (timepoint 2), or 49 mm^2^ (timepoint 3). Flank tumors and respective tdLNs were collected from ipsilateral inguinal LN for the heterotopic sites. For the orthotopic sites, oral cavity tumors and tdLNs were collected from the ipsilateral cervical LN. Harvested tissues were processed into single-cell suspensions. Tissue dissociation was followed as previously described [26]. Once in single-cell suspension, cells were counted, and one million cells were plated in U-bottom 96-well plates. Cells were stained with a comprehensive 28-color immune cell marker panel using fluorescent-conjugated antibodies. The fluorescent-conjugated antibodies used for flow cytometry are shown in Table S1. Flow cytometric data were acquired on a Cytek Aurora flow cytometer using SpectroFlo 3.2.1 software (Cytek, Fremont, CA, USA).

### 2.4. Flow Cytometry Analysis

Flow cytometric data were analyzed using FlowJo 10.10.0 software (FlowJo, Ashland, OR, USA). The gating strategy of the flow cytometric analysis of myeloid and lymphoid cell subsets is provided in Figure S1 and followed previous literature [27,28].

To visualize the immune cell populations within the 49 mm^2^ heterotopic (flank) and orthotopic (oral cavity) tumors, we performed t-distributed Stochastic Neighbor Embedding (tSNE) analysis of the 2 × 10^4^ CD45^+^ immune cells concatenated from each sample. Through immune cell marker visualization and manual gating, we identified several clusters in the tSNE plots representing CD8^+^ and CD4^+^ T-cells, natural killer (NK) cells, NK T-cells, dendritic cells (DCs), macrophages, monocytic-myeloid-derived suppressor cells (M-MDSCs), and granulocytic-MDSC (G-MDSCs).

### 2.5. Statistical Analysis

Differences in survival between the heterotopic and orthotopic ROC1 tumor models were evaluated with the Kaplan–Meier log-rank test. Flow analysis data were compared by two-way ANOVA with Tukey’s multiple comparisons test across the models. Multiple unpaired *t*-tests were conducted on tSNE data. All statistical analyses were performed using GraphPad Prism 10.6.1 software (GraphPad, San Diego, CA, USA). *p* values of less than 0.05 indicated a statistically significant difference.

## 3. Results

### 3.1. ROC1 Heterotopic and Orthotopic Tumor Growth Kinetics and Survival

**Figure 1:**
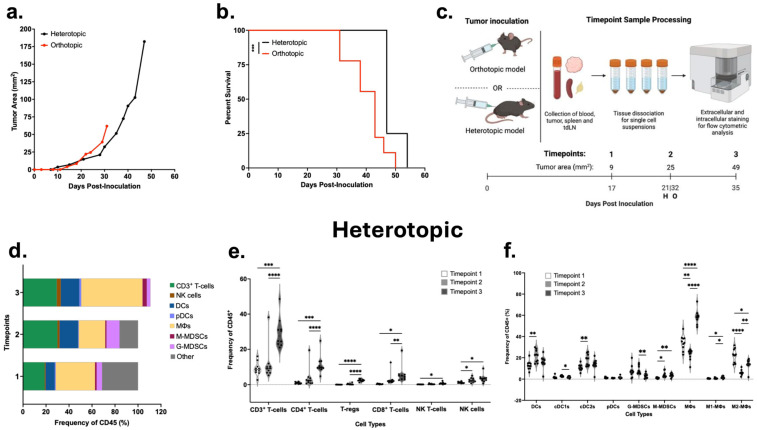

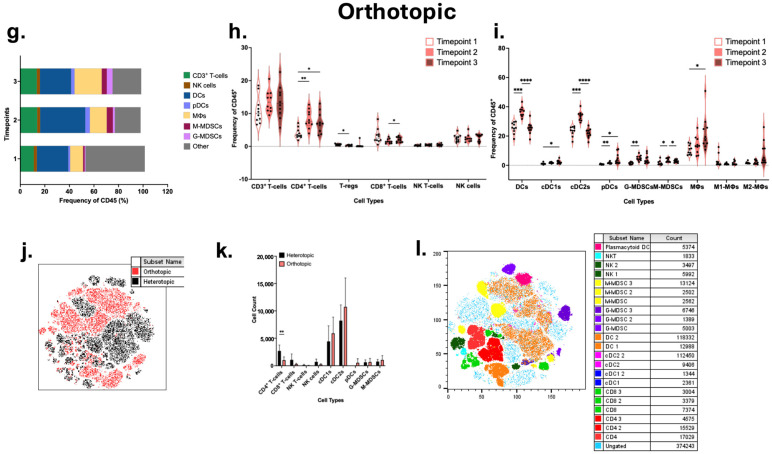
ROC1 heterotopic and orthotopic tumor growth kinetics, survival and immune composition in wild-type C57BL/6 mice. Mean tumor growth in heterotopic (black, *n* = 7) and orthotopic (red, *n* = 10) ROC1 tumor models. (**a**) Mean tumor growth data until the first mouse from each group was euthanized. (**b**) Survival of heterotopic and orthotopic models. Significance was evaluated by Kaplan–Meier Log-rank; *** Heterotopic vs. orthotopic, *p* < 0.0002. (**c**) Schematic of tumor inoculation and timeline of the three timepoints with their respective tumor area for sample processing in both models. Average heterotopic model (**d**) TIME composition and (**e**) lymphoid and (**f**) myeloid cells at the three timepoints (*n* = 10/timepoint). Average orthotopic model (**g**) TIME composition and (**h**) lymphoid and (**i**) myeloid cells at the three timepoints (*n* = 10/timepoint). Two-way ANOVA with Tukey’s multiple comparisons was conducted, * *p* < 0.05, ** *p* < 0.002, *** *p* < 0.0002, **** *p* < 0.0001. (**j**) Combined tSNE of the heterotopic and orthotopic tumors. (**k**) TIME composition from tSNE data represented in a bar graph. Multiple unpaired *t*-tests were conducted; ** CD4^+^ T-cells in heterotopic vs. orthotopic, *p* < 0.001. (**l**) tSNE of lymphoid cells in the heterotopic and orthotopic tumor models.

ROC1 flank (heterotopic model) and oral cavity (orthotopic model) tumor growth kinetics and survival were monitored to determine if there were site-specific differences. The tumor onset in the heterotopic model started at 10 days post-inoculation, while the orthotopic tumors began to develop at 14 days post-inoculation (Figure 1a). Overall, similar tumor growth kinetics were observed in both models. Tumors were not palpable until 10 days post-inoculation, grew slowly around 21 days post-inoculation, and then entered an exponential growth phase around 35 days post-inoculation. The heterotopic model had extended survival compared to the orthotopic model (Figure 1b). These differences in survival can be attributed to the euthanasia criteria being no larger than 15 mm in diameter in the heterotopic model compared to no larger than 12 mm in diameter in the orthotopic model. No differences in survival were observed between the two models when the euthanasia cut-off was set to 12 mm in diameter for analysis (Figure S2a). As expected, tumor progression resulted in increased tumor weights over time, with a significant difference in weight between late-stage heterotopic and orthotopic tumors (Figure S2b). To understand whether adaptive immunity played a role in the delayed tumor growth kinetics seen up to timepoint 2, we inoculated Rag1^−/−^ knockout mice with ROC1 tumor cells in the flank or oral cavity. Rag1^−/−^ knockout mice and C57BL/6 mice had similar tumor growth curves and survival in the heterotopic models (Figure S2c,d) and in the orthotopic models (Figure S2e,f). Together, these findings indicate that differences in tumor growth and survival are similar across the models and early tumor growth delay is not driven by adaptive immunity in this context, prompting a deeper examination of the immune landscape as tumors progress.

### 3.2. TIME Composition of ROC1 Heterotopic and Orthotopic Tumors Across Stages of Tumor Progression

To evaluate the tumor immune composition across the models as tumors progress, we took three timepoints: a small tumor at approximately 9 mm^2^ on day 17 post-inoculation (timepoint 1), a mid-size tumor at approximately 25 mm^2^ on day 21 for the heterotopic model or on day 32 for the orthotopic model (timepoint 2), or a large tumor at approximately 49 mm^2^ on day 35 post-inoculation (timepoint 3) (Figure 1c). Over time, the heterotopic TIME composition steadily increased in CD3^+^ T-cells, natural killer (NK) cells, dendritic cells (DCs), plasmacytoid DCs (pDCs), and monocytic myeloid-derived suppressor cells (M-MDSCs) (Figure 1d). However, granulocytic-MDSCs (G-MDSCs), which remained stable in frequency between timepoint 1 to timepoint 2, subsequently decreased from 8.31% to 3.17% by timepoint 3. Conversely, macrophage composition reduced from 34.30% to 22.99% by timepoint 2 but recovered to 58.52% upon timepoint 3. We further evaluated the immune cell subpopulations to understand the immunosuppression versus anti-tumor immune activity occurring in the TIME. In the heterotopic TIME, T-cell and NK cell populations had overall increases over time across all subtypes, including CD4^+^ T-cells, its subset of regulatory T-cells (T-regs), and NK T-cells (Figure 1e). This coincides with increases in DCs, specifically conventional DC type 2 (cDC2), from 10.86% to 18.22% between timepoints 1 and 2 (Figure 1f). This DC subset is important for priming of CD4^+^ T-cells. In the heterotopic TIME, G-MDSCs are stable from timpoint 1 to timepoint 2 but decrease from 8.31% to 3.17% by timepoint 3. M-MDSCs, on the other hand, increase from 1.11% at timepoint 1 to 4.27% at timepoint 2 and reduce to 3.65% at timepoint 3. Finally, overall macrophage populations, specifically M2-like macrophages, were significantly reduced from 22.23% to 6.18% between timepoint 1 and timepoint 2 and recovered to 13.96% by timepoint 3. The increasing trend in lymphoid cell populations suggests a surge in tumor-infiltrating lymphocytes (TILs) prepared for anti-tumor activity, and the presence of immunosuppressive cell types likely contributed to the loss of tumor growth control. Indeed, in addition to the presence of T-regs, increases in pro-tumor myeloid cell populations, such as M-MDSCs and M2-like macrophages, suggest a role in countering potential anti-tumor activity from T-cells or NK cells.

In the orthotopic site, we also observed increased numbers of CD3^+^ T-cells, macrophages, G-MDSCs, and M-MDSCs (Figure 1g). From timepoint 1 to timepoint 2, there was an increase in DCs from 25.52% to 36.69% and pDCs from 0.71% to 1.66%. From timepoint 2 to timepoint 3, there was a decrease in DCs from 36.69% to 25.38%. Notably, while the changes in these different cell populations varied over time, the overall TIME composition at both sites consisted primarily of macrophages, DCs and CD3^+^ T-cells. These findings show that, at all three timepoints, the TIME reflects a dynamic balance between immunosuppressive and anti-tumor responses, which ultimately shifts in favor of immunosuppression. Similarly to the heterotopic TIME, in the orthotopic TIME, we noted CD4^+^ T-cells increased as tumors progressed (Figure 1h). Interestingly, there was a decrease in T-regs from 0.55% at timepoint 1 to 0.29% at timepoint 2, indicating there was a shift in the anti-tumor activity in the TIME. This anti-tumor activity shift can be linked to the CD8^+^ T-cells increasing from 1.58% to 2.04% between timepoint 2 to 3. NK T-cells increased from 0.28% at timepoint 1 to 0.45% at timepoint 3, but this difference was not significant. Additionally, NK cells remained constant over time. Among the DCs, the conventional DC type 1 (cDC1) showed a significant increase from 1.15% at timepoint 1 to 2.30% at timepoint 3 (Figure 1i). However, the DCs were primarily cDC2s, as in the heterotopic tumor, and displayed similar trends, which may explain the increase in CD4^+^ T-cells. Plasmacytoid DCs increased from 0.71% at timepoint 1 to 4.63% at timepoint 3. Macrophages had a decrease in M1-like polarization over time, but this difference was not significant. The macrophages were primarily M2-like and increased over the timepoints. The presence of M2-like macrophage polarization indicates a tumor-promoting microenvironment. M-MDSCs and G-MDSCs increased from timepoint 1 to timepoint 2. M-MDSCs then decreased from timepoint 2 to timepoint 3. This suggests that MDSCs contribute to tumor progression during the early stages, but their influence becomes less significant at later stages. Despite differences in implantation site, both models showed a high proportion of CD3^+^ T-cells, DCs, and macrophages.

### 3.3. Late-Stage ROC1 Heterotopic Tumors Contain Higher Proportion of CD4^+^ T-Cells Than Orthotopic Tumors

As previously observed, there was a significant difference in weight between late-stage heterotopic and orthotopic tumors, which may be a confounding factor in a statistical comparison between the models (Figure S2b). Therefore, tSNE analysis was used to select a representative sample of the CD45^+^ immune cells concatenated from each tumor model at timepoint 3 to visualize the immune cell populations within 49 mm^2^ heterotopic and orthotopic tumors. Through immune cell marker visualization and manual gating, we identified several clusters in the tSNE plots representing CD8^+^ and CD4^+^ T-cells, NK cells, NK T-cells, DCs, macrophages, M-MDSCs, and G-MDSCs (Figure 1j). The only significant difference observed was the heterotopic model having a higher number of CD4^+^ T-cells than the orthotopic model (Figure 1k). Several cell types were identified based on their lineage and respective expression of functional markers (Figure 1l). When we compared the CD45^+^ frequency across the two models, we noted the heterotopic model had a significantly higher proportion of overall T-cells compared to the orthotopic model, at 29.31% versus 13.42%, respectively (Figure S3a). This is further emphasized when comparing the ratio of T-regs to CD8^+^ T-cells of both models, where this ratio is significantly higher in heterotopic at 0.558 versus the orthotopic tumors at 0.097 (Figure S3b). When comparing CD45^+^ frequency across the two models, the orthotopic model showed a significantly higher proportion of DCs, specifically the cDC2 subtype at 21.85%, compared to the heterotopic model, which showed 15.03% (Figure S3c). Additionally, while macrophages were a notable portion of the respective TIME in both models, macrophages made up 58.52% of the heterotopic site compared to 22.14% in the orthotopic site. These findings suggest that the tumor’s anatomical location influences the type of immune microenvironment it will develop, underscoring the importance of examining not only immune cell presence but also their functional state.

### 3.4. Reduction in Cytotoxic Markers in T-Cells and Increased Immune Checkpoint Stimulation in DCs, MDSCs and Macrophages Are Evidence of an Immunosuppressive TIME

**Figure 2:**
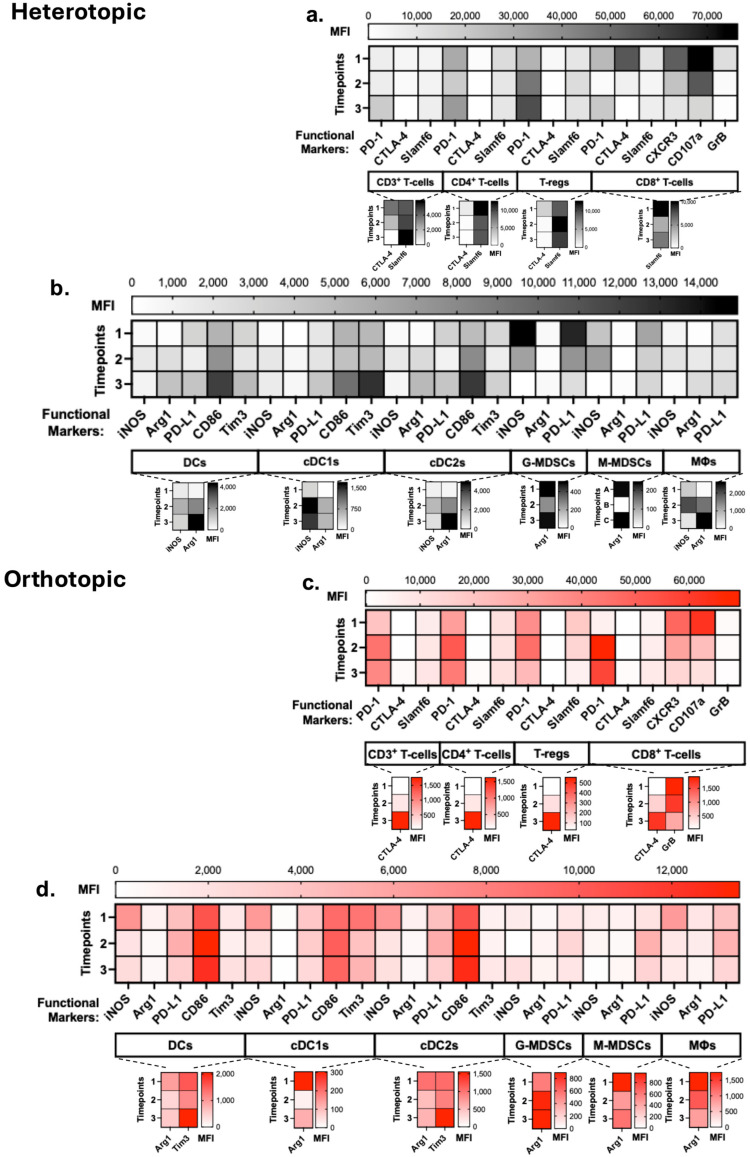
Tumor progression is accompanied by diminished T-cell cytolytic markers and an increase in immunosuppressive cell states in tumor-bearing wild-type C57BL/6. Heterotopic model (**a**) T-cell functional markers (PD-1, CTLA-4, Slamf6) and activation markers (CXCR3, CD107a, GrB) across the three timepoints (*n* = 10/timepoint). Scale indicates mean fluorescent intensity (MFI) of that respective marker. For both models, functional markers with relatively low MFI dynamic range are presented as insets, indicated by the dotted lines below their parent populations. Heterotopic model functional markers and activation from (**b**) DCs, MDSCs and macrophages (MΦs) at the three timepoints. Orthotopic model (**c**) T-cell functional and activation markers across the three timepoints (*n* = 10/timepoint). Orthotopic model functional markers and activation from (**d**) DCs, MDSCs and macrophages (MΦs) at the three timepoints. Statistical analyses performed using two-way ANOVA with Tukey’s multiple comparisons.

To further understand the contributions of heterotopic and orthotopic TIME compositions to immunosuppression that leads to tumor progression, we investigated the activation and functional states of these lineage cell populations within each model. Significant increases in frequencies over time were observed across all T-cell subpopulations in the heterotopic TIME. However, these cells increasingly expressed the exhaustion marker PD-1 (Figure 2a). In CD8^+^ T-cells, CTLA-4 was expressed at timepoint 1. Expression of this early activation marker was, however, reduced significantly over time. CXCR3, a chemokine receptor that is a marker for type 1 immunity effector response and important for cell trafficking to tissue, had reduced expression over time in CD8^+^ T-cells present in the tumor. Additional markers of CD8^+^ T-cell cytotoxic activation, CD107a and Granzyme B (GrB), similarly decreased over time. Notably, we observed high CD107a expression at timepoint 1, suggesting early cytotoxic degranulation functionality, which was ultimately lost. Together, these results indicate early activation and cytotoxic function and, ultimately, impaired anti-tumor immunity that contributed to tumor progression. Analysis of myeloid cell populations in the heterotopic TIME revealed an increased CD86 expression, an indicator of DC activation, was observed across these subtypes (Figure 2b). Additionally, increased Tim3 and PD-L1 expression involves the impairment of DC antigen presentation and effective anti-tumor T-cell function. Inducible nitric oxide synthase (iNOS) and Arginase 1 (Arg1) are indicators of MDSC and macrophage suppressive functions driving T-cell inhibition. Across these cell types, particularly in macrophages, iNOS expression was reduced, while Arg1 increased. PD-L1 expression in MDSCs was simultaneously reduced over time, suggesting weakened checkpoint-mediated suppression.

The orthotopic lymphoid cell states showed predominantly PD-1 expression in CD4^+^ and CD8^+^ T-cells (Figure 2c). CTLA-4 and Slamf6 were expressed by CD4^+^ and CD8^+^ T-cells across the timepoints. As for activation markers, we observed that CXCR3 expression in CD8^+^ T-cells was highest at timepoint 1, with a gradual decrease by timepoint 3. Downregulation of CXCR3 can lead to decreased CD8^+^ T-cell tumor infiltration. At timepoint 1, CD8^+^ T-cells had high expression of CD107a and GrB but ultimately decreased expression of both markers over time, indicating decreased cytolytic activity. The dual capacity of DCs to either enhance anti-tumor immunity or promote immunosuppression is determined by their functional state. iNOS expression in DCs and M-MDSCs was at the highest at timepoint 1 and decreased over time, indicating other immunosuppression mechanisms occurring in the TIME as tumors progressed (Figure 2d). There were no significant differences across timepoints in Arg1 expression in DCs, G-MDSCs, M-MDSCs, and macrophages. PD-L1 levels remained highly expressed in DCs, M-MDSCs, and macrophages, which may be another method of immunosuppression inflicted on effector cells that inhibits their cytolytic activity. As for activation markers, DCs chronically expressed CD86 but ultimately were limited in their antigen presentation properties by the expression of PD-L1. Additionally, Tim3 expression in DCs decreased from timepoint 1 to timepoint 2. These findings suggest a pro-tumor immune microenvironment as tumors progressed by the decrease in cytolytic activity in T-cells and increase in immunosuppressive cell states in macrophages, M-MDSCs, and DCs.

### 3.5. Systemic Immune Response in ROC1 Heterotopic and Orthotopic Tumors Indicates Preserved Effector Cell Reserves Despite Immune Suppressive Immunocyte Trafficking

**Figure 3:**
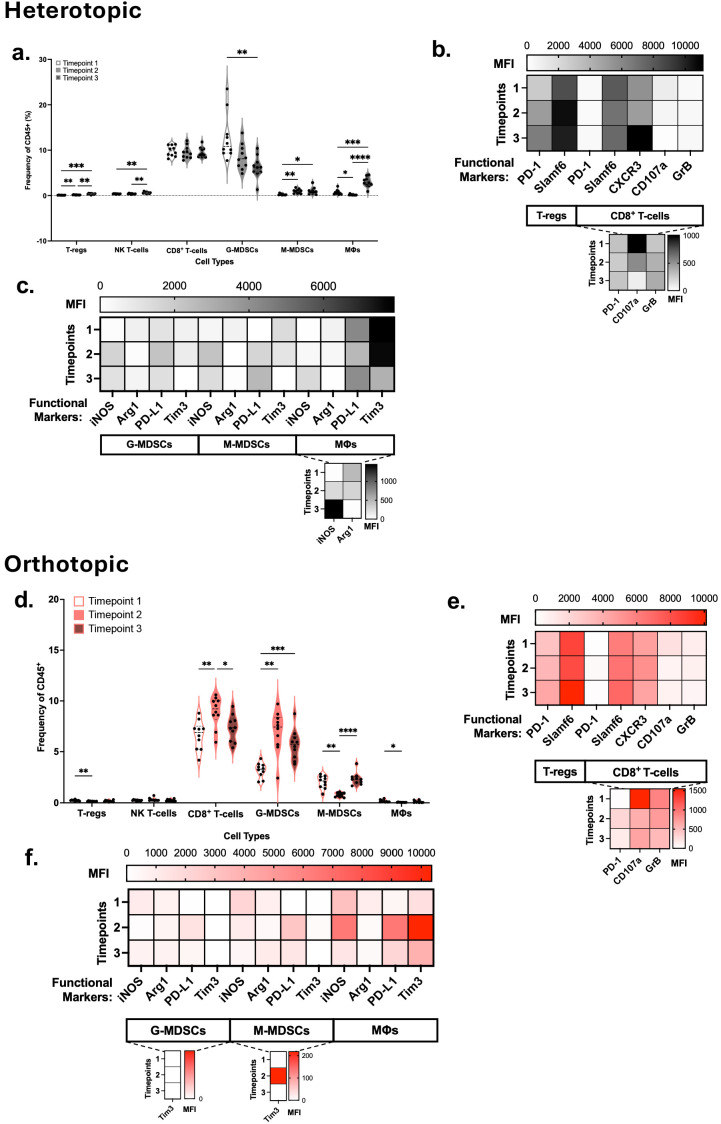
ROC1 tumor progression marked by a decline in circulating functional TILs and an increase in suppressive cell types in tumor-bearing wild-type C57BL/6. (**a**) T-regs, NK T-cells, CD8^+^ T-cells, G-MDSCs and M-MDSCs in the blood of ROC1 tumor-bearing heterotopic wild-type mice (*n* = 10/timepoint). Expression of functional and activation markers in (**b**) lymphoid cells and (**c**) myeloid cells in the ROC1 heterotopic model. Scale indicates mean fluorescent intensity (MFI) of that respective marker. For both models, functional markers with relatively low MFI dynamic range are presented as insets, indicated by the dotted lines below their parent populations. (**d**) Blood composition of immune cells in ROC1 orthotopic tumor model (*n* = 10/timepoint). Expression of functional and activation markers in (**e**) lymphoid cells and (**f**) myeloid cells in the orthotopic model. Statistical analyses were performed using two-way ANOVA with Tukey’s multiple comparisons, * *p* < 0.05, ** *p* < 0.002, *** *p* < 0.0002, **** *p* < 0.0001.

To assess the immunosuppressive capabilities of tumor cells in the host, we examined the host systemic response. Blood and spleen were collected from heterotopic and orthotopic ROC1 tumor-bearing mice, and their immune profiles were compared across timepoints. In the peripheral blood of heterotopic tumor-bearing mice, T-regs and NK T-cells increased across all timepoints (Figure 3a). While circulating NK T-cells may indicate a degree of systemic immunity and readiness, T-regs in the blood may counter this and indicate dysregulation of trafficking to appropriate sites. Interestingly, frequencies of CD8^+^ T-cells in the blood remained consistent over time. In the investigation of myeloid cell populations, increased levels of circulating M-MDSCs and macrophages over time were observed. G-MDSCs decreased while remaining the highest immunosuppressive cell type in circulation. When investigating the functional state of the immune cells, we noted that circulating T-regs increasingly expressed PD-1 and Slamf6, indicating a state of activation and the potential for persistent suppression (Figure 3b). Moreover, CD8^+^ T-cells increasingly expressed CXCR3 while expressing low levels of CD107a and GrB, suggesting primed T-cells that may be marked for trafficking to the tumor but are not actively cytotoxic. M-MDSCs found in the blood increased their expression of iNOS and decreased Arg1 over time, suggesting a more inflammatory state for systemic immune activation rather than a localized suppressive response conditioned by the tumor (Figure 3c). Macrophages found in the blood increased over time while increasing expression of iNOS, while PD-L1 expression recovers, and Tim3 expression continues to decrease. PD-L1 expression in macrophages complement the observations present in the tumor.

In the peripheral blood of orthotopic tumor-bearing mice, T-regs decreased from timepoint 1 to 2, similar to observations in the TIME (Figure 3d). There were no significant differences in NK T-cell composition in the blood of orthotopic tumor-bearing mice across the timepoints. CD8^+^ T-cells increased from timepoint 1 to 2; however, a decrease was observed from timepoints 2 to 3. The increase in circulating T-cells indicates an initial immune response to the tumor, which ultimately drops as tumors progress. G-MDSCs in the blood experienced a similar increase from timepoint 1 to 2 and a decrease from 2 to 3 as the tumors progress. Interestingly, M-MDSCs in the blood were inversely proportional to the tumor composition, indicating M-MDSCs were recruited to the TIME from the blood. There was a decrease in macrophage composition from timepoint 1 to 2. In comparing the CD45^+^ frequencies across the two models, we noted that the heterotopic model had a significantly higher proportion of circulating T-cell populations, namely CD8^+^ T-cells, compared to the orthotopic model (Figure S4). Conversely, M-MDSCs were found in the blood of orthotopic tumor-bearing mice at approximately double the frequency compared to those with heterotopic tumors. T-regs increasingly expressed PD-1 and Slamf6 across the timepoints (Figure 3e). The circulating CD8^+^ T-cells highly expressed CXCR3 across tumor progression, indicating they may be migrating into the tumor site to conduct their effector function. CD107a and GrB were highly expressed in CD8^+^ T-cells at timepoint 1. Over time, CD107a expression decreased in CD8^+^ T-cells in the blood, following a similar trend as seen in the TIME. Meanwhile, PD-1 and Slamf6 expression increased in the CD8^+^ T-cells. G-MDSCs iNOS expression was highest at timepoint 1 and then decreased at timepoint 2, indicating other immunosuppressive mechanisms occurring as tumors progressed (Figure 3f). Immunosuppressive potential of circulating G-MDSCs was observed with the increase in PD-L1 expression at timepoint 2 and continuous Arg1 expression. While Tim3 was not significantly expressed by G-MDSCs, in M-MDSCs, Tim3 expression was upregulated at timepoint 2. Similar to the tumor, iNOS expression in M-MDSCs was highest at timepoint 1 and decreased over time. Like G-MDSCs, there was upregulation of PD-L1 at timepoint 2 and Arg1 expression at timepoint 3. Macrophages also observed an increase in PD-L1 and Tim3 expression at timepoint 2. We next evaluated whether immunosuppression was also present in peripheral tissues.

**Figure 4:**
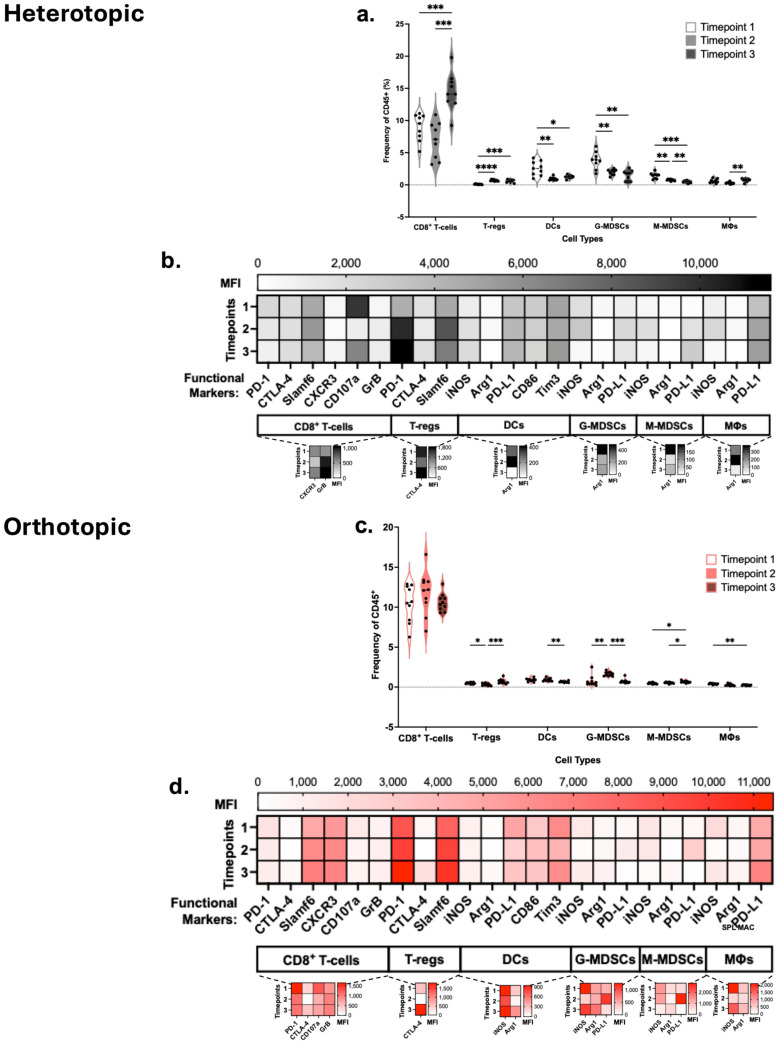
Systemic suppression through MDSCs and macrophages in the ROC1 tumor-bearing spleens of wild-type C57BL/6. (**a**) CD8^+^ T-cells, DCs, G-MDSCs, M-MDSCs, and macrophages (MΦs) in the spleens of heterotopic tumor-bearing mice (*n* = 10/timepoint). (**b**) Functional markers of CD8^+^ T-cells, T-regs, DCs, G-MDSCs, M-MDSCs, and MΦs in the heterotopic model. Scale indicates mean fluorescent intensity (MFI) of that respective marker. For both models, functional markers with relatively low MFI dynamic range are presented as insets, indicated by the dotted lines below their parent populations. (**c**) Immune composition in spleens of orthotopic tumor-bearing mice (*n* = 10/timepoint). (**d**) Functional markers in the orthotopic model. Significance was evaluated by two-way ANOVA with Tukey’s multiple comparisons, * *p* < 0.05, ** *p* < 0.002, *** *p* < 0.0002, **** *p* < 0.0001.

Spleens from ROC1 heterotopic tumor-bearing mice showed an increase in all T-cell subtypes, most notably CD8^+^ T-cells, over time as tumors grew (Figure 4a). DCs and G-MDSCs decreased in the spleen, while M-MDSCs and macrophages were present in low frequencies. When examining the functional state of the immune cells, both PD-1 and CTLA-4 checkpoint markers were steadily expressed by T-cell subtypes, suggesting early activation and persistent stimulation (Figure 4b). CD8^+^ T-cells also increased their CXCR3 expression, which is associated with trafficking. Notably, we observed an overall increase in CD107a expression, with a reduction at timepoint 2, suggesting the trafficking of these cells out of the spleen. Our observations suggest a possible T-cell expansion in the spleen due to an active immune response and readiness for migration. PD-L1, CD86 and Tim3 expression remained persistent in DCs. In MDSC and macrophage populations, iNOS expression initially increased from timepoint 1 to timepoint 2 and reduced by timepoint 3, while PD-L1 expression increased across the timepoints.

In spleens from orthotopic tumor-bearing mice, there were no significant differences in CD8^+^ T-cell frequency across the timepoints (Figure 4c). DC composition decreased from timepoint 2 to 3, indicating the loss of circulating DCs. This also aligns with the decrease in DCs seen in the tumor at timepoint 3. Similar to the observations seen in blood, G-MDSCs in the spleen increased from timepoint 1 to 2 and then decreased from timepoint 2 to 3. M-MDSC composition increased in the spleen over time, indicating the increase in systemic M-MDSCs. Comparison of the CD45^+^ frequency across the two models indicated that CD8^+^ T-cells were present in the spleen in higher amounts in heterotopic versus orthotopic tumor-bearing mice (Figure S5). Similar to the orthotopic tumor and the spleen of heterotopic tumor-bearing mice, PD-L1, Tim3, and CD86 were chronically expressed by DCs (Figure 4d). iNOS expression in M-MDSCs, G-MDSCs, and macrophages was higher at timepoint 1. Meanwhile, Arg1 expression was higher at later timepoints in the MDSC populations. Similar to the tumor and blood, PD-L1 expression in G-MDSCs and M-MDSCs was higher at timepoint 2. Macrophage PD-L1 expression was high, especially at timepoint 3. PD-L1 expression in myeloid subsets from the spleens of both tumor models suggests the potential for suppression of T-cells by contributing to inhibition of cytotoxic cells, T-reg expansion, and reduced T-cell priming.

### 3.6. tdLNs Show a Pattern of Immunosuppressive Immunocyte Infiltration

**Figure 5:**
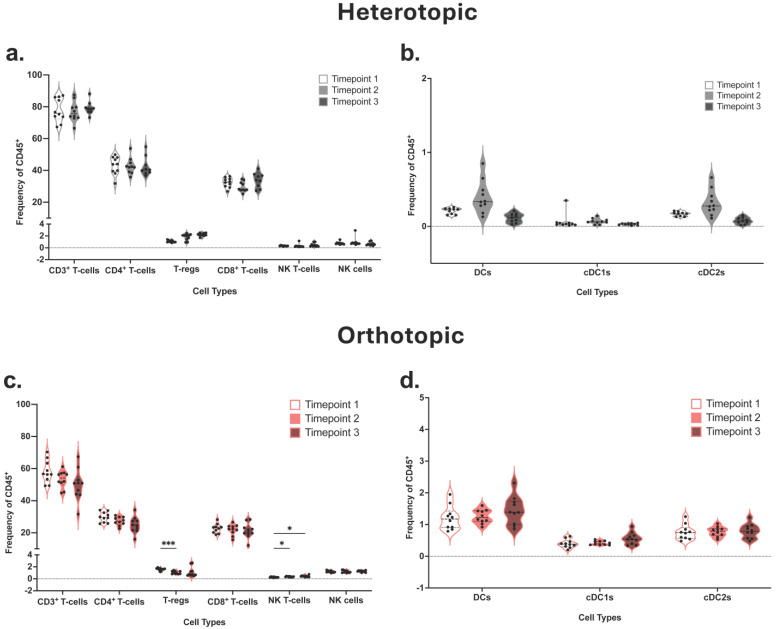
Tumor-draining lymph nodes (tdLNs) of heterotopic and orthotopic ROC1 tumor models show similar overall immune cell compositions in wild-type C57BL/6. Heterotopic (**a**) lymphoid and (**b**) myeloid composition of tdLNs (*n* = 10/timepoint). Orthotopic (**c**) lymphoid and (**d**) myeloid composition of tdLNs (*n* = 10/timepoint). Statistical analyses were performed using two-way ANOVA with Tukey’s multiple comparisons, * *p* < 0.05, *** *p* < 0.0002.

To further determine the role of immune cell recruitment in cancer progression, we focused on the response of the host’s immune system in the tdLNs. tdLNs from tumor-bearing mice in both heterotopic and orthotopic models were collected. In the heterotopic model, CD8^+^ T-cells increased from timepoint 2 to 3, although not significantly, while DCs, specifically cDC2 cells, increased during the early tumor growth phase but subsequently decreased as the tumor reached timepoint 3 (Figure 5a,b). These population modifications mirror changes in the heterotopic TIME and are indicative of a normal immune response. That is, following exposure to tumor antigen, these immune cell populations may initially expand or be recruited, respectively, but ultimately are suppressed, leading to a halt in expansion and trafficking to the tdLNs. Other populations, including NK cells, remained steady over time. CD3^+^ T-cells, including CD4^+^ and CD8^+^ T-cells, decreased in the orthotopic tumor model tdLNs across the timepoints (Figure 5c). A significant decrease in T-regs was observed from timepoint 1 to 2, similar to the TIME. NK T-cells increased significantly across the timepoints; this difference was also observed in the TIME, but it was not significant. Both NK cell and DC composition in the tdLNs remained constant (Figure 5c,d). tdLNs associated with late-stage heterotopic tumors had higher overall frequencies across T-cell populations, specifically CD4^+^ and CD8^+^ T-cells, in comparison with those of the orthotopic model (Figure S6a). Late-stage tdLN associated with the orthotopic site, however, resulted in significant increases across DC populations, specifically in both cDC1 and cDC2 subtypes, compared to the heterotopic model (Figure S6b).

**Figure 6:**
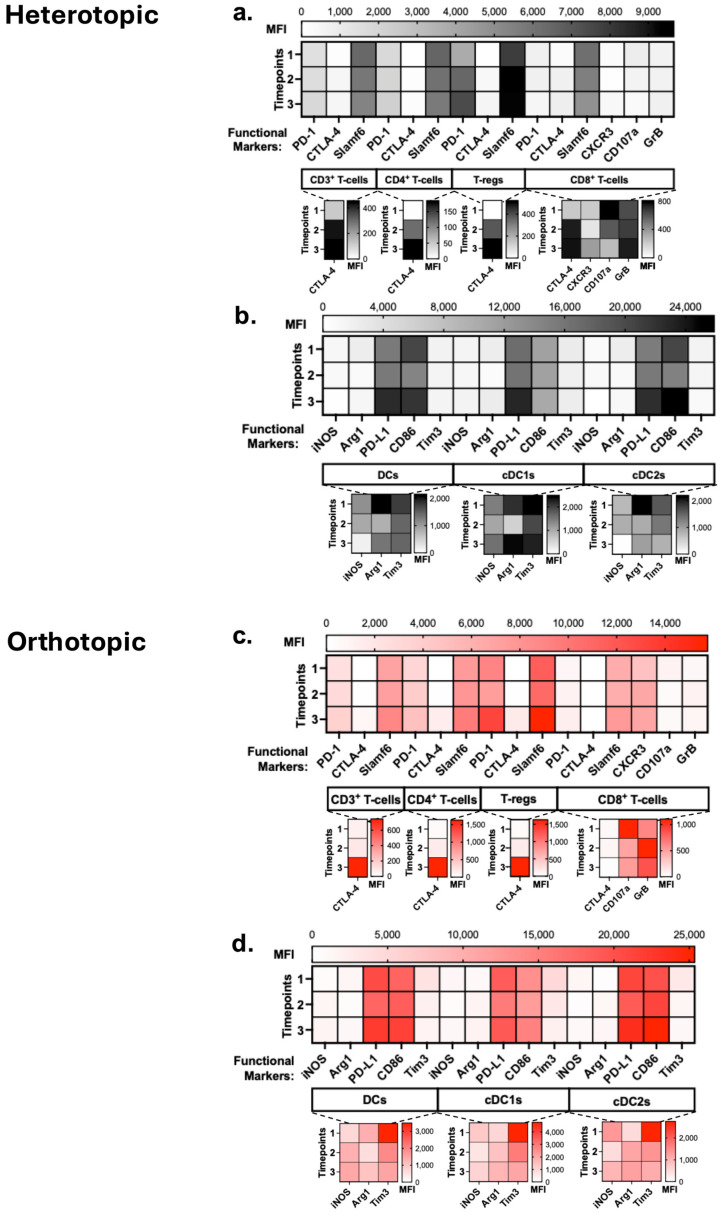
Functional markers of immune cells in the tumor-draining lymph nodes potentiate immunosuppressive conditions in wild-type C57BL/6. Heterotopic tdLNs functional markers in (**a**) lymphoid and (**b**) myeloid cells (*n* = 10/timepoint). Scale indicates mean fluorescent intensity (MFI) of that respective marker. For both models, functional markers with relatively low MFI dynamic range are presented as insets, indicated by the dotted lines below their parent populations. Orthotopic tdLNs functional markers in (**c**) lymphoid and (**d**) myeloid cells (*n* = 10/timepoint). Statistical analyses were performed using two-way ANOVA with Tukey’s multiple comparisons.

To understand the lymphoid and myeloid cell states, we compared the expression of functional markers in the tdLNs. In the heterotopic tdLN, we observed slight decreases in Slamf6 expression over time, with consistently moderate PD-1 and low CTLA-4 expression in T-cells (Figure 6a). The converse is true, however, in T-regs, which had striking increases in PD-1 and Slamf6 over time. CD8^+^ T-cells also showed a notable increase in CXCR3 expression while decreasing CD107a by timepoint 3. Together, these results mark increased suppression by T-regs, while CD4^+^ T-cells have weakened immune activity and CD8^+^ T-cells are primed for trafficking but not actively cytotoxic. No major differences in iNOS and Arg1 expression were observed in DCs (Figure 6b). PD-L1 and CD86 were highly expressed in DCs. PD-1, CTLA-4 and Slamf6 expression was highest at timepoint 3 in CD4^+^ T-cells in orthotopic tdLNs (Figure 6c). While PD-1 expression was highest at timepoint 3, CTLA-4 and Slamf6 expression in CD8^+^ T-cells remained constant. CXCR3 in CD8^+^ T-cells increased from timepoint 1 to timepoint 2. CD107a was highest in timepoint 1, and GrB was highest in timepoint 2 in CD8^+^ T-cells, indicating the early stages of cytolytic activity. As with heterotopic tumor-associated tdLNs, DCs showed no major differences in iNOS and Arg1 expression (Figure 6d). Additionally, PD-L1 and CD86 expression was high in DCs, with the highest expression at timepoint 3. Lastly, Tim3 expression in the tdLNs was highest in early-stage tumors at timepoint 1.

## 4. Discussion

Despite advances in surgery, radiation, and systemic therapy, outcomes for many patients with head and neck squamous cell carcinoma remain poor, particularly in HPV-negative oral cancer, where resistance to immunotherapy and high recurrence rates continue to limit durable responses. These therapeutic challenges underscore the need for preclinical models that faithfully recapitulate the human tumor immune microenvironment and more accurately predict clinical efficacy.

Syngeneic murine models are widely used in cancer research to evaluate immunotherapeutic strategies within an intact, functional immune system and to interrogate dynamic interactions within the TIME. In head and neck cancer research, emerging therapies are frequently assessed using subcutaneous heterotopic (flank) models because they are technically straightforward, allow convenient longitudinal tumor measurements [9], and provide direct access for localized interventions such as radiation therapy [10,11]. However, these practical advantages come at the cost of anatomical and immunologic relevance. Orthotopic models, in which tumors are established within the native organ site, are increasingly regarded as more translationally representative, as they better mirror the human TIME and more reliably predict therapeutic responses [12,13,14,15,16]. Accordingly, there is growing effort to develop and characterize orthotopic HNSCC models suitable for preclinical therapeutic testing [17,18,19,20]. Prior studies have also sought to delineate differences in the TIME across murine HNSCC models, highlighting the importance of tumor location in shaping immune composition and function [21,22,23].

Herein, we utilized ROC1, an HPV-negative murine oral cancer cell line derived from carcinogen exposure. ROC1 represents a clinically relevant model for studying tobacco-related, immunotherapy-refractory disease, as the ROC1 model has mutational patterns reflective of tobacco-associated human oral squamous cell carcinoma and exhibits resistance to conventional immunotherapy [24,25]. In this study, we assessed tumor growth kinetics and survival and performed longitudinal, multi-compartment immune profiling of ROC1 tumors implanted in both heterotopic and orthotopic sites. We systematically analyzed tumors, tumor-draining lymph nodes, peripheral blood, and spleen at three defined timepoints to capture the temporal evolution of local and systemic immune responses. By directly comparing the TIME between orthotopic and heterotopic tumors, we aimed to determine how anatomical context influences immune architecture and to guide rational model selection for studying specific immunopathologic mechanisms or evaluating immunotherapies in head and neck cancer.

We compared ROC1 tumor growth and survival in heterotopic and orthotopic models to assess site-specific differences in model establishment. Tumors were detectable earlier in the heterotopic model than in the orthotopic model; however, overall growth kinetics were similar. Although the heterotopic model initially showed extended survival, this difference was attributable to distinct euthanasia criteria. When a uniform 12 mm endpoint was applied, no survival differences were observed, indicating comparable biological behavior between models. Tumor weights increased over time in both models, with higher late-stage weights in the heterotopic group, likely reflecting differences in allowable tumor burden and anatomical constraints [29,30]. The ROC1 tumor growth kinetics in the Rag1^−/−^ mice were similar to those in the wild-type C57BL/6 mice from each respective model, confirming that an appropriately functioning adaptive immune system was not primarily responsible for restraining early tumor growth. This effect likely reflects both insufficient initial effector B-cell and T-cell responses and the potent suppressive activity of T-regs, MDSCs, or M2-like macrophages, which may act concurrently to promote tumor progression. Additionally, innate immune cells such as NK cells can destroy cancer cells through their cytolytic activity [31]. Future work should consider eliminating NK cells from the TIME to determine whether the delayed tumor growth is caused by these cells. The immune cells in both heterotopic and orthotopic tumors, such as DCs, macrophages, and CD3^+^ T-cells, can play dual roles in the immunosuppressive TIME. Specifically, for T-cells, we noted across both models that there was an increased expression of immune checkpoints, notably PD-1, in CD4^+^ and CD8^+^ T-cells as tumors progressed. Furthermore, the cytolytic activity of CD8^+^ T-cells was affected by a decrease in functional markers such as CD107a and GrB. Increased expression of immune checkpoints and a decrease in functional markers indicated dysfunctional TILs as tumors progressed. Additionally, a decrease in expression of chemokine receptor CXCR3 was noted in CD8^+^ T-cells. CXCR3 is important for recruiting T-cells and enhancing the interaction of T-cells with antigen presenting cells [32,33].

To further drive immunosuppression, myeloid subsets were observed to express Arg1. The increasing expression of Arg1 is a mechanism MDSCs use to suppress T-cell function by metabolizing L-Arginine [34]. Additionally, M-MDSCs had an increase in PD-L1 expression. The upregulation of PD-L1 is a mechanism of immune evasion MDSCs use to inhibit the cytolytic activity of T-cells by binding to PD-1 receptors on T-cells [35]. Also, increased expression of PD-L1 in DCs was found in the TIME and is known to hinder antigen presentation. Peng et al. found that, by blocking PD-L1 in established tumors, it promotes re-activation of tumor-infiltrating T-cells [36]. While there were activated cDC1s as indicated by an increase in CD86 at late-stage heterotopic tumors, there was also an increase in Tim3 in DCs at late stages. De Mingo Pulido et al. found that targeting Tim3 in intratumoral CD103 DCs improves chemotherapy responses in breast cancer [37]. While anti-Tim3 would enhance cDC1 functionality, MDSCs can also express Tim3 and can be targeted by the immune checkpoint inhibition. Future efforts should focus on targeting the immune checkpoint blockade to desired effector immune cells while minimizing unintended activation of immunosuppressive cell populations.

At early tumor stages, G-MDSCs and M-MDSCs appear to be the primary drivers of immunosuppression through iNOS and Arg1 expression. In contrast, in late-stage tumors, our data suggests that M2-like macrophages become the dominant immunosuppressive population in both heterotopic and orthotopic models. Also, across multiple myeloid subsets—including macrophages—upregulation of PD-L1 represents one of several mechanisms by which M2 macrophages mediate immune suppression. In heterotopic tumors, a portion of F4/80^+^ macrophages are not categorized as either M1- or M2-like. This population is, instead, marked by iNOS^−^MHC class II^hi^ expression and is indicative of reduced inflammatory potential in an intermediate population of tumor-associated macrophages (TAMs). As tumors progress, these cells transition to MHC class II^lo^ M2-like suppressive TAMs [38]. This corresponds to the observations of macrophages in this study. By timepoint 3, this iNOS^−^MHC class II^hi^ population is increasingly present in the tumor, suggesting corresponding tumor tolerance in an ongoing move toward M2-like TAM classification. The DC composition in the TIME consists largely of the cDC2 subtype, which allows for strategic targeting and prediction of therapeutic response. For instance, in a study of a patient cohort with whole HNSCC tumors with a high expression signature of the 15-gene hypoxia-associated gene signature, high frequencies of cDC2 were found to be predictive of high locoregional recurrence and distant metastasis [39].

When we compared the late-stage tumors in both models, we noted that the heterotopic model had higher lymphoid cell subsets than the orthotopic model. In our tSNE analysis, we found the key difference between both models was in the heterotopic model, which contained more CD4^+^ T-cells compared to the orthotopic model at late stages. As in this and other studies of orthotopic HNSCC models, we found that the ROC1 heterotopic tumor model contained relatively high frequencies of TILs, which have been associated with improved survival in patients with HNSCC [40,41,42,43,44,45]. Interestingly, ROC1 heterotopic tumors notably even had T-cell frequencies consistently higher than those of the orthotopic site. Comparisons of T-reg to CD8^+^ T-cell ratios in advanced tumors showed notably more T-regs in heterotopic versus orthotopic tumors, suggesting site-specific importance of T-regs. However, together, this highlights the importance of comprehensive TIME profiling and consideration of functional states. Loss of both CD107a and GrB over the course of tumor progression, for example, suggests that the T-cells present in the tumor, particularly of the orthotopic site where more CD8^+^ T-cells are present compared to T-regs, are not active to complete potential effector function. The presence of T-cells alone is not enough to drive anti-tumor response.

To assess the systemic impact of tumor growth on myeloid populations, we analyzed immune composition in peripheral tissues. Additionally, stable or increasing peripheral expression of Slamf6 expression in CD8^+^ T-cells and T-regs in both heterotopic and orthotopic models suggests an ability for critical renewal of the T-cell pool [46]. Between the heterotopic and orthotopic site, CD8^+^ T-cells in blood were present at higher frequences in heterotopic tumor-bearing mice with advanced disease. Despite T-cell expansion and increased CXCR3 expression, loss of CD107a and GrB expression again suggests that these T-cells are not functionally active and are further suppressed by PD-1-expressing T-regs. At late-stage disease, we also observed a sustained increase in MDSCs in the blood of mice bearing either heterotopic or orthotopic tumors. Jordan et al. found that G-MDSCs accumulate in the spleens of cancer patients, similar to what occurs in tumor-bearing mice, and can ultimately predict outcomes in cancer patients [47]. Additionally, in the orthotopic site specifically, M-MDSC composition increased in the spleen over time, indicating the increase in systemic M-MDSCs, compared to the heterotopic site. Patients with tumors infiltrated by MDSCs typically have a poor prognosis [48]. However, MDSCs could serve as a potential therapeutic target for cancer treatment. For instance, in a phase 1b trial (NCT02124850), patients with HNSCC were treated with a TLR8 agonist, motolimod, and cetuximab preoperatively, and their tumors had less MDSCs and increased M1 monocytes [49].

Studying the tumor and lymph node microenvironments is essential to understanding immune dysfunction and suppression. In patients with head and neck cancer, lymph node metastasis actively remodels lymph node architecture, creating organized immunosuppressive niches composed of myeloid cells and cancer-associated fibroblasts [50]. These niches promote T-cell dysfunction, activation of T-regs, and overall are an active driver of systemic immunosuppression that supports metastatic tolerance and progression. Consistent with systemic observations of dysfunctional T-cell expansion and upregulation of CXCR3 expression in the blood and spleen, as well as in the tdLNs, the heterotopic model TIME indicates an influx of T-cell infiltration as tumors progress, demonstrating the capacity of T-cell recruitment to the tumor site. Moreover, in observation of late-stage tumor-draining lymphatics, the heterotopic tdLN does not contain dendritic cells that would contribute to priming of these cells. Meanwhile, the orthotopic model had an increase in DCs in the tdLN compared to the heterotopic model, indicating antigen presentation capabilities occurring. Kono et al. treated MOC1 oral tumors established in the buccal mucosa, as well as the subcutaneous flank, with an anti-PD-1 blockade and demonstrated efficacy in the orthotopic MOC1 tumor due to cDC1s’ greater antigen uptake in the tdLNs and enhanced generation of antigen-specific T-cells relative to flank tdLNs [23]. Similarly, in the mEER HPV-positive tongue cancer model, there was protective immunity following immune checkpoint inhibitor treatment [21]. We recently demonstrated that administration of a cyclic dinucleotide (CDN), a known activator of the stimulator of interferon genes pathway in dendritic cells, loaded in a biomaterial system in combination with anti-PD-1 and anti-CTLA-4 in ROC1 orthotopic tumor-bearing mice results in extended survival and partial tumor responses [51]. This indicates that, while DCs are necessary for antigen presentation and while checkpoint blockade can restore T-cell functionality, the immunosuppressive mechanisms present in the ROC1 model still need to be addressed for complete responses. In the present study, the tdLNs had DC populations with high PD-L1 expression, and the expression of CD86 in these populations increased as tumors grew. This indicates that, although antigen presentation was occurring, the DCs eventually became dysfunctional. These findings indicate the need to analyze both the tumor and lymph node microenvironments to understand cancer evasion and determine potential treatment options for preclinical models.

There is increasing consensus that understanding of TIME composition is important to understanding immune dynamics and treatment response across cancer types. Indeed, researchers have previously examined the location-based differences in the TIME of HNSCC preclinical models, often at a single timepoint. An exception to this was a research group that studied the tumor and tdLN of a single site, the MOC1 model of the buccal mucosa, to understand the immune cell composition over the course of tumor progression at two timepoints and to predict potential immunotherapy responses [22]. Our findings are the first to broadly analyze local and systemic immunity across tumor, tdLNs, blood, and spleen of two different engraftment sites, heterotopic and orthotopic ROC1 models, by flow cytometry to compare the TIME and host response at three distinct timepoints across tumor progression. Previous studies of the ROC1 model used multiplex immunohistochemistry to immunophenotype only late-stage ROC1 tongue tumors [24]. The earliest we conducted our analysis was at the first palpable tumor at 9 mm^2^. Future work should closely examine the spatial interactions between effector and suppressive cell types in the tdLNs and TIME to improve our understanding of these models and the role the host immune system plays in the cancer setting. Furthermore, this study comprehensively demonstrates the nuances in the dynamics of cellular composition and functional states, both locally and systemically, associated with tumor progression at both heterotopic and orthotopic tumor sites. These results show that disease progression is generally characterized by dysfunctional T-cells, immunosuppressive regulatory T-cells, and myeloid populations. These different immune compositions of tumors at early and late timepoints should further inform model selection and potential treatment strategies.

## 5. Conclusions

In conclusion, this study establishes the orthotopic and heterotopic ROC1 model as a clinically relevant platform for investigating immune dynamics in HPV-negative, tobacco-associated head and neck cancer. Through longitudinal, multi-compartment immune profiling, we demonstrate that tumor progression is accompanied by coordinated local and systemic immunosuppression. Our results highlight distinct temporal shifts in dominant immunosuppressive mechanisms, with MDSCs shaping early-stage suppression and M2-like macrophages and checkpoint upregulation reinforcing dysfunction at later stages. Importantly, parallel alterations in tumors and tumor-draining lymph nodes underscore the necessity of evaluating both sites to accurately model therapeutic responses. Collectively, this work provides a comprehensive framework for selecting appropriate models and for designing rational immunotherapeutic strategies in head and neck cancer.

## Data Availability

The data that support the findings of this study are available upon request from the corresponding author.

## Supplementary Materials

The following supporting information can be downloaded at: https://www.mdpi.com/article/10.3390/cancers18101607/s1, Table S1. Myeloid and lymphoid panel immune cell markers with their respective fluorophores, clones, catalog numbers, and vendors; Figure S1. The flow cytometry gating strategy for CD45+ populations; Figure S2. Heterotopic (flank) and Orthotopic (oral cavity) tumor models do not depend on adaptive immune system for delayed tumor growth; Figure S3. Comparing the late-stage heterotopic (flank) and orthotopic (oral cavity) tumor models immune composition in wild-type C57BL/6; Figure S4. Reduction in circulating T-cells in heterotopic (flank) tumors in wild-type C57BL/6; Figure S5. Comparing the late-stage heterotopic (flank) and orthotopic (oral cavity) tumor models spleen immune composition in wild-type C57BL/6; Figure S6. Comparing the late-stage heterotopic (flank) and orthotopic (oral cavity) tumor models tdLN immune composition in wild-type C57BL/6.

## Abbreviations

The following abbreviations are used in this manuscript:

Arg1	Arginase 1
CD103	Cluster of differentiation 103
CD107a	Cluster of differentiation 107a
CD3^+^ T-cell	Cluster of differentiation 3-positive T-cell
CD4^+^ T-cell	Cluster of differentiation 4-positive T-cell
CD45	Cluster of differentiation 45
CD8^+^ T-cell	Cluster of differentiation 8-positive T-cell
CD86	Cluster of differentiation 86
cDC1	Conventional dendritic cell type 1
cDC2	Conventional dendritic cell type 2
CTLA-4	Cytotoxic T-lymphocyte-associated protein 4
CXCR3	Chemokine receptor 3
DC	Dendritic cell
G-MDSC	Granulocytic-myeloid-derived suppressor cell
GrB	Granzyme B
HNSCC	Head and neck squamous cell carcinoma
iNOS	Inducible nitric oxide synthase
L/D	Live/dead
LN	Lymph node
MDSC	Myeloid-derived suppressor cell
M-MDSC	Monocytic-myeloid-derived suppressor cell
mEER	Mouse E6 E7 Ras
MOC1	Murine oral cancer 1
NK cell	Natural killer cell
NK T-cell	Natural Killer T-cell
PD-1	Programmed cell death protein 1
PD-L1	Programmed cell death ligand 1
pDC	Plasmacytoid dendritic cell
Slamf6	SLAM Family Member 6
T-reg	Regulatory T-cell
tdLN	Tumor-draining lymph node
TIL	Tumor-infiltrating lymphocyte
Tim3	T-cell immunoglobulin and mucin domain 3
TIME	Tumor immune microenvironment
tSNE	t-distributed stochastic neighbor embedding
