# Supplementary material for: Tumor Implantation Site of Syngeneic Oral Cancer Models Differentially Induces Site-Dependent Local and Systemic Immunosuppression
Source: Cancers (Basel). 2026 May 15;18(10):1607. doi: 10.3390/cancers18101607 (PMC13205123; doi:10.3390/cancers18101607)
Supplement: Supplementary file 1 [file cancers-18-01607-s001.zip › cancers-4267753-supplementary.pdf]

Tumor Implantation Site of Syngeneic Oral Cancer Models Differentially Induces Site-Dependent Local and Systemic Immunosuppression

Andrea H. Molina[1†], Gemalene M. Sunga[1,2†], Shawn Nguyen[1], Neeraja Dharmaraj[1], Ratna Veeramachaneni[2], Roberto Rangel[2], Jeffrey N. Myers[2], Jeffrey D. Hartgerink[3,4], Andrew G. Sikora[2], Simon Young[1*]

[1]Katz Department of Oral and Maxillofacial Surgery, School of Dentistry, The University of Texas Health Sci-ence Center at Houston, Houston, TX 77054, USA

[2]Department of Head and Neck Surgery, The University of Texas MD Anderson Cancer Center, Houston, TX 77030, USA

[3]Department of Chemistry, Rice University, 6100 Main Street, Houston, Texas 77098

[4]Department of Bioengineering, Rice University, 6100 Main Street, Houston, Texas 77098

[†]These authors contributed equally.

*Corresponding author.

Supplemental Figures and Tables

Table S1. Myeloid and lymphoid panel immune cell markers with their respective fluorophores, clones, catalog numbers, and vendors.

Marker (Fluorophore)	Clone	Catalog #	Vendor
Arg1 (BUV805)	A1exF5	368-3697-82	Invitrogen
CD103 (BV510)	M290	563087	BD
CD107a (BV421)	1D4B	121617	Biolegend
CD11b (BV650)	M1/70	101259	Biolegend
CD11c (AF488)	N418	117311	Biolegend
CD3 (violetFluor500)	17A2	85-0032-U100	Cytek/Tonbo
CD4 (BUV661)	RM4-4	612974	BD
CD45 (BUV395)	30-F11	564279	BD
CD8a (BB515)	53-6.7	564422	BD
CD86 (BV750)	GL1	747439	BD
CTLA4 (APC R700)	UC10-4F10-11	565778	BD
CXCR3 (PE/Dazzle594)	S18001A	155914	Biolegend
EPCAM (BUV737)	G8.8	741818	BD
F4-80 (eFluor450)	BM8	48-4801-82	Invitrogen
FoxP3 (AF647)	MF-14	126408	Biolegend
GrB (PE/Cy7)	NGZB	25-8898-82	Invitrogen
iNOS (APC)	CXNFT	17-5920-82	Invitrogen
L/D (Live/Dead Blue)		L34962	Invitrogen
Ly6C (PerCP-Cy5.5)	HK1.4	45-5932-82	Invitrogen
Ly6G (PerCPeF710)	1A8	46-9668-82	Thermo/eBio
MHC II (APC-Cy7)	M5/114.15.2	25-5321-U100	Cytek/Tonbo
NK1.1 (PE/Cy5)	PK136	108716	Biolegend
PD-1 (PE)	J43	50-9985-U100	Cytek/Tonbo
PD-L1 (BV605)	10F-9G2	124321	Biolegend
PDCA-1 (BV711)	927	127039	Biolegend
Slamf6 (BUV615)	13G3	751341	BD
Tim3 (PE/Fire810)	RMT3-23	119745	Biolegend
XCR1 (BV785)	ZET	148225	Biolegend

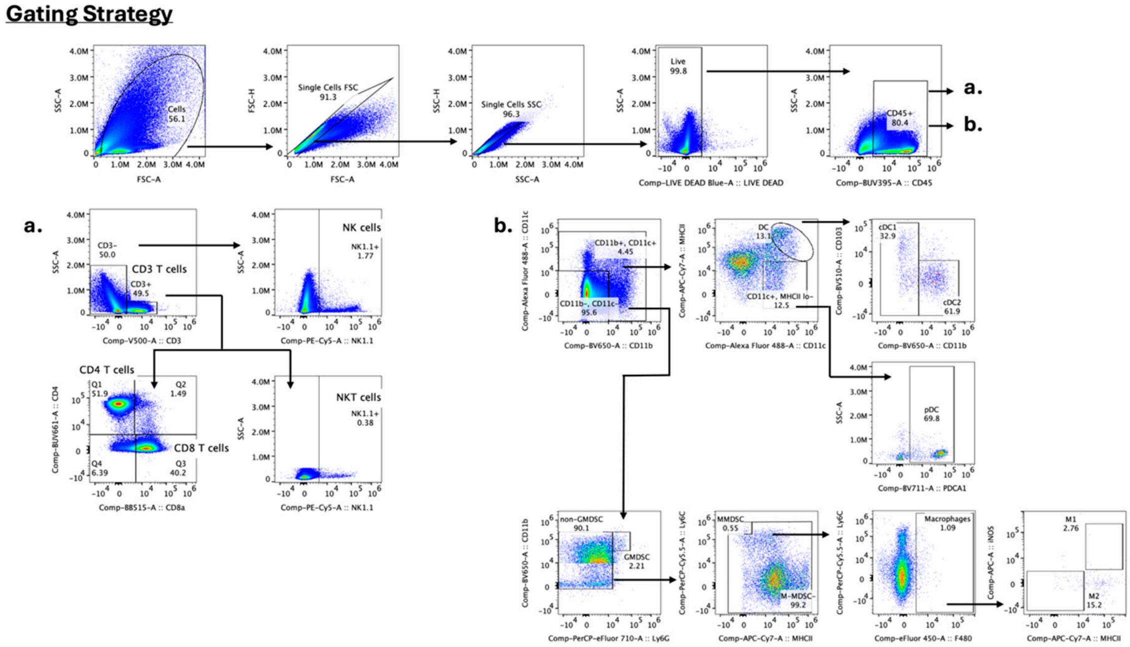

Figure S1. **The flow cytometry gating strategy for CD45$^+$ populations.** The (**a**) lymphoid and (**b**) myeloid cells.

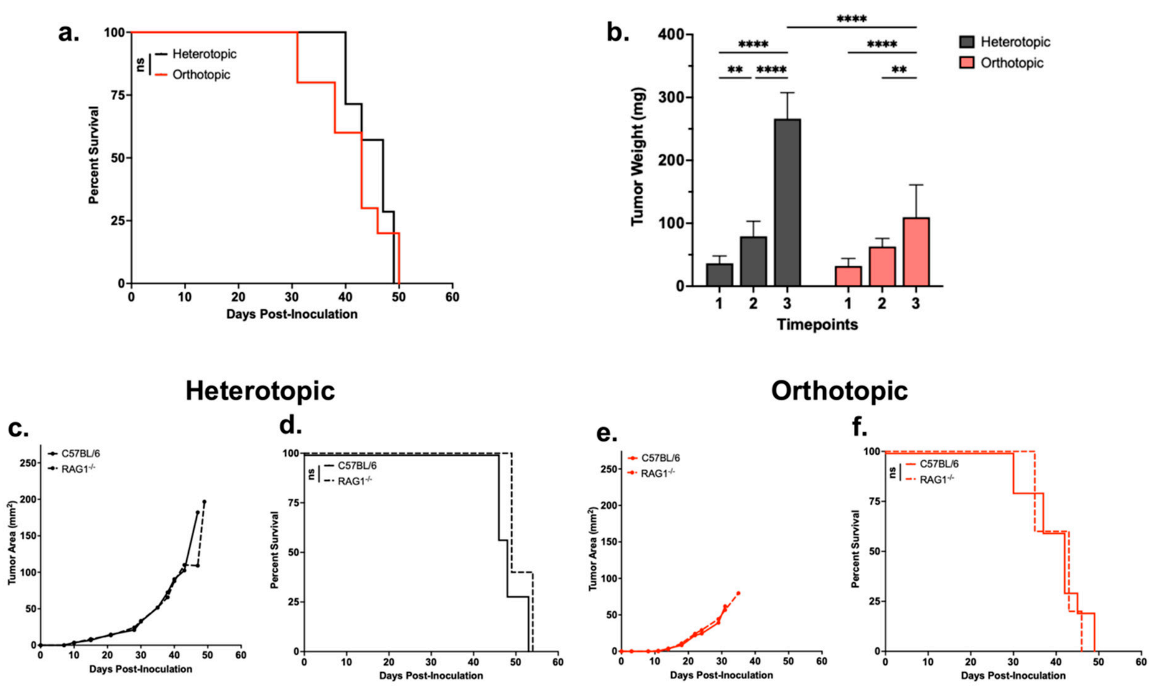

Figure S2. **Heterotopic (flank) and Orthotopic (oral cavity) tumor models do not depend on adaptive immune system for delayed tumor growth.** (**a**) Comparison of the survival curves of the ROC1 heterotopic and orthotopic tumor models in wild-type C57BL/6 mice. Euthanasia criteria set when tumors reach a size of 12 mm in diameter. Significance was evaluated by Kaplan-Meier Log-rank, ns: not significant. (**b**) Measurements of tumor weight in heterotopic and orthotopic ROC1 tumors in wild-type C57BL/6 mice. Two-way ANOVA with Tukey's multiple comparisons were conducted, *p < 0.05, **p < 0.002, ***p < 0.0002, ****p < 0.0001. Heterotopic ROC1 tumors in wild-type C57BL/6 and RAG1$^{-/-}$ mice (**c**) mean tumor growth and (**d**) survival curve. Mean tumor growth shown until the first mouse from each group is euthanized. Orthotopic ROC1 tumors in wild-type C57BL/6 and RAG1$^{-/-}$ mice (**e**) mean tumor growth and (**f**) survival curve.

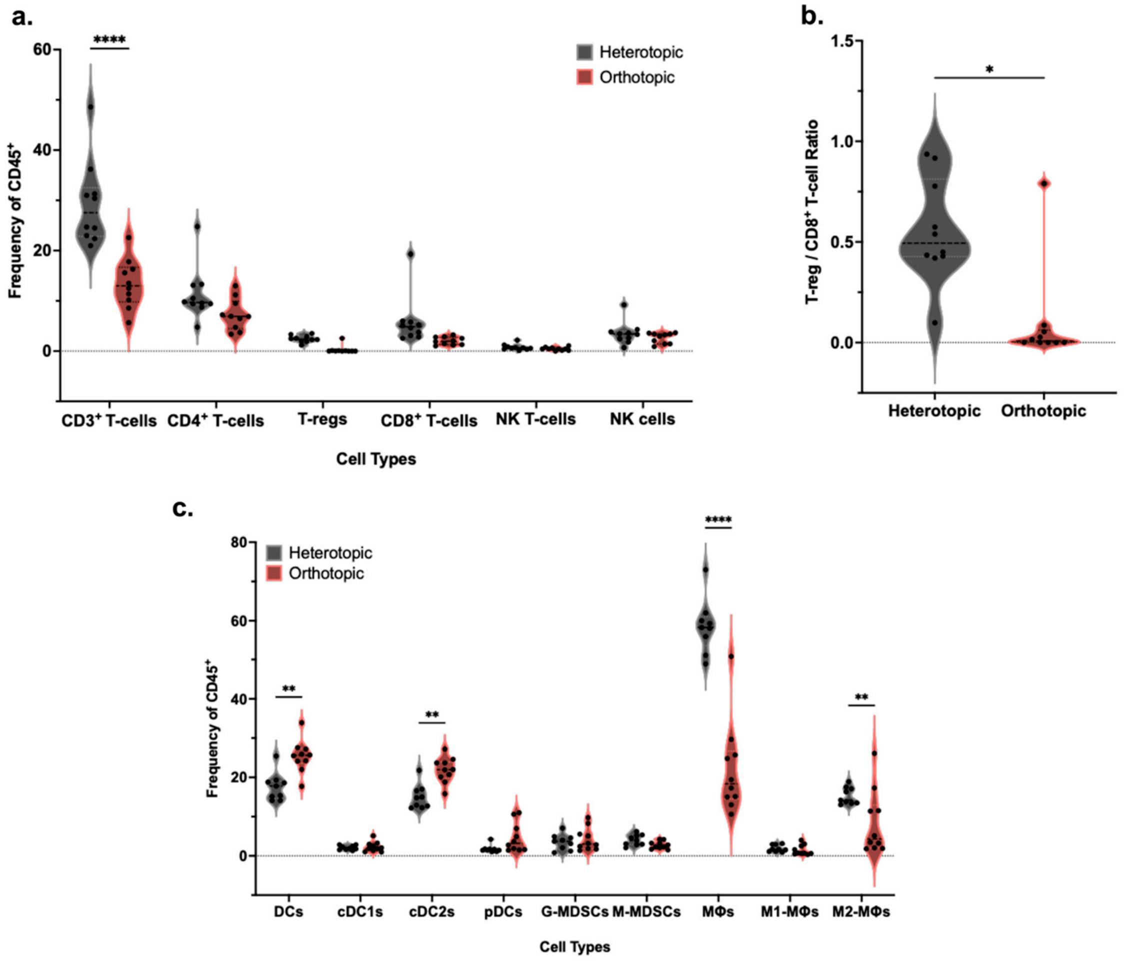

Figure S3. **Comparing the late-stage heterotopic (flank) and orthotopic (oral cavity) tumor models immune composition in wild-type C57BL/6.** (**a**) Frequency of $CD45^+$ in the lymphoid compartment. (**b**) Ratio of regulatory to $CD8^+$ T-cells. (**c**) Frequency of $CD45^+$ in myeloid compartment.

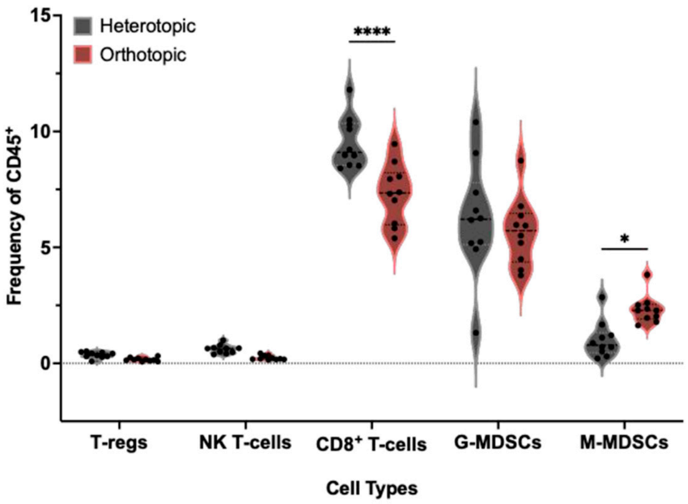

Figure S4. Reduction in circulating T-cells in heterotopic (flank) tumors in wild-type C57BL/6. Comparison of T-cell and MDSC subtype frequencies of $CD45^{+}$ cells in peripheral blood of heterotopic and orthotopic ROC1 tumor-bearing wild-type C57BL/6 mice.

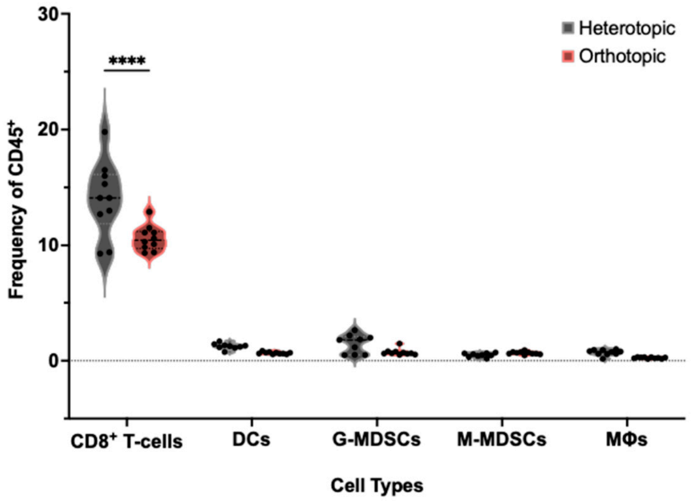

Figure S5. Comparing the late-stage heterotopic (flank) and orthotopic (oral cavity) tumor models spleen immune composition in wild-type C57BL/6. $CD8^{+}$ effector T-cell and myeloid cell subtype frequencies of $CD45^{+}$ cells.

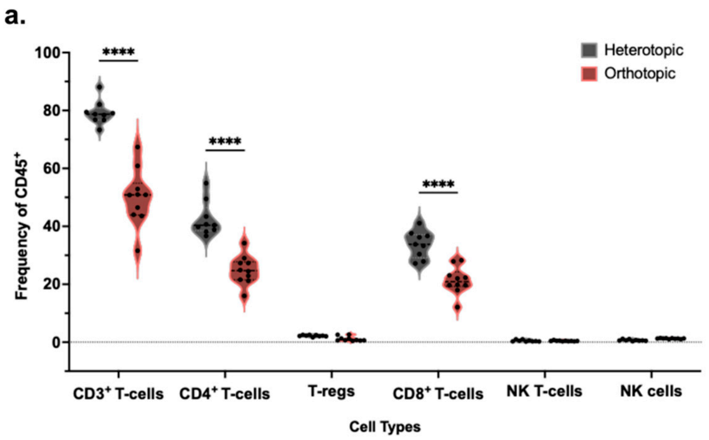

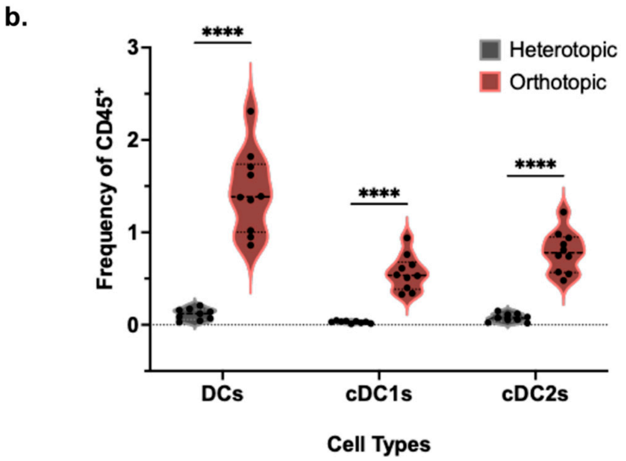

Figure S6. Comparing the late-stage heterotopic (flank) and orthotopic (oral cavity) tumor models tdLN immune composition in wild-type C57BL/6. Frequency of $CD45^+$ in (**a**) lymphoid and (**b**) myeloid cells.
